# Supplementary material for: The relationship between sitting balance, trunk control and mobility with predictive for current mobility level in survivors of sub-acute stroke
Source: PLoS One. 2021 Aug 5;16(8):e0251977. doi: 10.1371/journal.pone.0251977 (PMC8341576; doi:10.1371/journal.pone.0251977)
Supplement: Attachment — Submitted filename: review comments.docx [file pone.0251977.s001.docx]

Review_PONE-D-20-25714

Major comments

#１. What is the significance of using trunk/sitting balance performance to predict mobility?

Please explain in the introduction. While trunk performance and/or sitting balance are important to mobility from previous research, I don’t think that as a reason to exclude other factors (e.g. lower extremity strength). What does reporting moderate predictive validity as a result of limited predictors lead to? Please also highlight the significance of this entire study in the introduction.

#2. Most importantly, I don’t see the clinical significance of predicting current TUG in a patient with measurable TUG. What is important is not predicting TUG, but that the mobility to be assessed by TUG can be explained a lot by sitting balance?

3. The two objectives seem to create confusion. What you want to clarify is the correlation between TUG and other performance test? Or is it trunk/sitting performance as a predictor of TUG? The conclusions do not seem to emphasize the correlation.

4. Although your study is a cross-sectional design, there are several representations that can be used to predict future mobility. Please give appropriate consideration to the information available from the results.

5. What is the significance or hypothesis of the MBI that was evaluated? Do you just want to investigate the correlation to TUG? ADLs were discussed in the previous studies presented in the introduction, but they were not mentioned in the DISCUSSION.

Minor comments

overall

#1. In the title, aim, and abstract, you use “among sitting balance, trunk control, mobility, and activities daily living" and "between sitting balance, trunk control, mobility and ADL”, it's hard to understand what you want to compare them to.

The results only show an correlation between mobility and other factors.

This can lead to a misunderstanding and a misguided purpose, as if it investigated a correlation among all factors.

#2. The key word was “chronic stroke”, is that correct?

abstract

#3. P2, L2. Is it gait ability or mobility that you predict? If you don't distinguish and define it, then please unify.

#4. P2, L10. Is the Finding notation TCT (r = -0.65)? There should be a space before and after “=”.

#5. P2, L11-14. Please indicate what the ROC curve or AUC is intended to do. to predict mobility?

#6. P2, L17. “...predicting the improvement of mobility after stroke.” does not prove improvement from this cross-sectional design.

Introduction

#7. P3, L10-30. The introduction as a whole hypothesizes the usefulness of SBS and these indicators as tools that can predict ADLs. Nevertheless, as a purpose, SBS is not the subject matter and what it predicts is not ADL. So, It’s hard to understand the flow of why you want to predict mobility. The connection from background to purpose seems somewhat forced. Please clarify the hypothesis and revise the structure.

#8. P2, L1-5. The use of the sitting balance tools scarce and BBS is explained at the beginning of the Introduction, but in the context of the “overall, SBS, Trunk Impairment Scale…(P3, L14-16)” there are other assessments that can be measured in the supine and sitting positions besides the SBS, Please explain these discrepancies.

#9. With regard to trunk control and sitting balance used in various contexts, it is often difficult to separate them. For example, the TCT and TIS also include sitting balance as an entry. Are there any definitions used in this study and any differences between there two elements?

Methods

#10. P4, L1-2. There is no mention of walking ability in the subject recruitment, is there a standard? At this time of year, I don't think everyone is capable of TUG. Or should readers understand that only those who are able to walk on advertisement have applied?

#11. P4, L3-4. Related to the above, what are the reasons for drop-outs in cross-sectional studies? Does this mean that there was a request from the patient, but it took a long time for the actual measurement to be taken, resulting in health deterioration and discharge?

#12. P4, L2. What is your rationale for using MMSE > 23 as the inclusion criteria? Does it appear that cognitive ability has a significant impact on the content of this assessment?

#13. P4, L13. Can you indicate the extent of “...experience in using these tools”? It seems ambiguous.

#14. P4, L20-24. Please indicate the original citation for the TUG at the beginning of the description of the TUG (Reference No. 14). In addition, please describe the speed conditions of the TUG.

#15. P4, L28-30. Please reiterate the reference of SBS reliability.

#16. P5, L12-13. Please reiterate the reference of TCT validity.

#17. P5, 34-35. What was the reason for comparing the two groups using only the Chi-square test after confirming the normal distribution in the statistical analysis?

Results

#18. P6, L20. SBS and TIS have similar numbers in the results. What are the correlation coefficients between these two variables?　Discussion also states that it is very closely related [2]. Even though the concepts are different, we get the impression that the content of the test is also partially common. It is recommended to emphasize the concept and relationship between the two variables in the introduction or discussion.

Discussion

#19. P6, L29-30. “The results of the present study...had significantly reduced mobility (TUG),” is natural because it generates two groups according to TUG. It is an unnecessary expression.

#20. P6, L29-34. Several repetitions of the results are described. Please summarize (especially the numbers) and organize them.

#21. P7, L11-13. The sentence of “Specifically, the stroke suvivors ... may not be included.”, what is your rationale for describing the inappropriate compensatory strategy and use assistive device? The authors don't consider the assistive device in recruitment and allow it to be used in the TUG, but what was the device usage rate?

#22. P7, L15-18. What do the authors mean by “However, in this study, ... postural control and mobility." It is somewhat difficult to understand the implications of these sentences.

Is there any evidence to be drawn from the results of this context or from previous studies?”

#23. P7, L19-23. The sentence of “The TCT and ... for TIS, respectively)”, this is the kind of sentence is a repetition of the results, or it should be written in the Results section.

#24. P7, L19. Please state the rationales or add a citation for “the TCT and PASS-TC are suitable for use in acute and subacute patients.”

#25. P7, L23-25. There is a lack of textual basis or citation for “In addition, the balance…required to perform the TUG.”

#26. P8, L4-6. What do these citations explain about “Medley et al and …examination of TIS [15, 18]”? Since SBS and TIS are newer reports than these articles, please clarify what you mean by these citations. Also, what do you mean by “in SBS, which is traditionally used,” in the sentence that follows?

#27. Please make the Limitation clear. As I see it, there are several limitations.

Table 1.

#28. What is the meaning of †?

#29. The percentages of the paralysis location are not shown.

Table 3.

#30. * does not appear to be in the table. Please remove it.

#31. Is this table showing a cut-off score to predict the mobility available group?

I hope that my comment is very useful for the improvement of the article.
