# Supplementary material for: The relationship between sitting balance, trunk control and mobility with predictive for current mobility level in survivors of sub-acute stroke
Source: PLoS One. 2021 Aug 5;16(8):e0251977. doi: 10.1371/journal.pone.0251977 (PMC8341576; doi:10.1371/journal.pone.0251977)
Supplement: Attachment — Submitted filename: Response to reviewers.docx [file pone.0251977.s002.docx]

**Reply to comments**

When submitting your revision, we need you to address these additional requirements.

1. Please ensure that your manuscript meets PLOS ONE's style requirements, including those for file naming. The PLOS ONE style templates can be found at

<https://journals.plos.org/plosone/s/file?id=wjVg/PLOSOne_formatting_sample_main_body.pdf> and

<https://journals.plos.org/plosone/s/file?id=ba62/PLOSOne_formatting_sample_title_authors_affiliations.pdf>

Reply: Thank you for your kind instruction. As per your comment, we have checked the PLOS ONE style and corrected the manuscript according to the style.

2. Please address the following:
- Please refer to any sample size calculations performed prior to participant recruitment. If these were not performed please justify the reasons. Please refer to our statistical reporting guidelines for assistance (<https://journals.plos.org/plosone/s/submission-guidelines.#loc-statistical-reporting>).
- Please include additional information regarding the survey or questionnaire used in the study and ensure that you have provided sufficient details that others could replicate the analyses. For instance, if you developed a questionnaire as part of this study and it is not under a copyright more restrictive than CC-BY, please include a copy, in both the original language and English, as Supporting Information.

Reply: Thank you for your valuable comment. As per your comment, we have calculated the sample size and added the description in the Methods section as follows.

“***Sample size calculation***

Using the G Power 3.1 statistical tool to achieve a statistical power of 80% with statistical significance at *p*<0.05 (two-tailed test) and an effect size of r=0.5, a total sample size of 26 participants was required for present study.”

3. During your revisions, please note that a simple title correction is required to ensure no errors of grammar: "Correlation between sitting balance, trunk control, mobility and daily living activities with predictive validity of mobility level in survivors of sub-acute stroke". Please ensure this is updated in the manuscript file and the online submission information.

Reply: Thank you for your valuable comment. As per your comment, we have revised the title as follows.

“**Correlation between mobility and sitting postural control, and predictive validity of for mobility level in survivors with sub-acute stroke**”

4.Thank you for stating the following financial disclosure:

 [No.].

 At this time, please address the following queries:

a. Please clarify the sources of funding (financial or material support) for your study. List the grants or organizations that supported your study, including funding received from your institution.
b. State what role the funders took in the study. If the funders had no role in your study, please state: “The funders had no role in study design, data collection and analysis, decision to publish, or preparation of the manuscript.”
c. If any authors received a salary from any of your funders, please state which authors and which funders.
d. If you did not receive any funding for this study, please state: “The authors received no specific funding for this work.”

Please include your amended statements within your cover letter; we will change the online submission form on your behalf.

Reply: Thank you for your kind instruction. As per your comment, we have added the descriptions as follows.

“**Financial disclosure**

The authors received no specific funding for this work.

**Competing Interests**

The authors have declared that no competing interests exist.

**Declaration of conflicting interests**

The author confirms that there is no conflict of interest.”

5. Thank you for stating the following in your Competing Interests section:

[No.].

Please complete your Competing Interests on the online submission form to state any Competing Interests. If you have no competing interests, please state "The authors have declared that no competing interests exist.", as detailed online in our guide for authors at http://journals.plos.org/plosone/s/submit-now

 This information should be included in your cover letter; we will change the online submission form on your behalf.

Please know it is PLOS ONE policy for corresponding authors to declare, on behalf of all authors, all potential competing interests for the purposes of transparency. PLOS defines a competing interest as anything that interferes with, or could reasonably be perceived as interfering with, the full and objective presentation, peer review, editorial decision-making, or publication of research or non-research articles submitted to one of the journals. Competing interests can be financial or non-financial, professional, or personal. Competing interests can arise in relationship to an organization or another person. Please follow this link to our website for more details on competing interests: http://journals.plos.org/plosone/s/competing-interests

Reply: Thank you for your kind instruction. As per your comment, we have added the descriptions as follows.

“**Financial disclosure**

The authors received no specific funding for this work.

**Competing Interests**

The authors have declared that no competing interests exist.

**Declaration of conflicting interests**

The author confirms that there is no conflict of interest.”

6. Please include your tables as part of your main manuscript and remove the individual files. Please note that supplementary tables (should remain/ be uploaded) as separate "supporting information" files

Reply: Thank you for your kind instruction. As per your comment, we have included the tables in the manuscript.

7. Your ethics statement should only appear in the Methods section of your manuscript. If your ethics statement is written in any section besides the Methods, please move it to the Methods section and delete it from any other section. Please ensure that your ethics statement is included in your manuscript, as the ethics statement entered into the online submission form will not be published alongside your manuscript.

Reply: Thank you for your kind instruction. As per your comment, we have described the ethic statement in the Methods section only.

Reviewers' comments:

Reviewer's Responses to Questions

Comments to the Author

1. Is the manuscript technically sound, and do the data support the conclusions?

The manuscript must describe a technically sound piece of scientific research with data that supports the conclusions. Experiments must have been conducted rigorously, with appropriate controls, replication, and sample sizes. The conclusions must be drawn appropriately based on the data presented.

Reviewer #1: Partly

Reviewer #2: Yes

Reply: Thank you for your valuable comment. As per your comment, we have revised the Conclusion section as follows.

“On the present study, the SBS showed the highest correlation for mobility using TUG in sub-acute stroke survivors. Also, SBS was revealed as the most appropriate examination tool for predicting the mobility by TUG in sub-acute stroke survivors. Through additional studies, it should be suggested about standardization to determine the superiority of sitting postural control.”

2. Has the statistical analysis been performed appropriately and rigorously?

Reviewer #1: Yes

Reviewer #2: Yes

Reply: Thank you for your decision.

3. Have the authors made all data underlying the findings in their manuscript fully available?

The [PLOS Data policy](http://www.plosone.org/static/policies.action#sharing) requires authors to make all data underlying the findings described in their manuscript fully available without restriction, with rare exception (please refer to the Data Availability Statement in the manuscript PDF file). The data should be provided as part of the manuscript or its supporting information, or deposited to a public repository. For example, in addition to summary statistics, the data points behind means, medians and variance measures should be available. If there are restrictions on publicly sharing data—e.g. participant privacy or use of data from a third party—those must be specified.

Reviewer #1: Yes

Reviewer #2: Yes

Reply: Thank you for your decision.

4. Is the manuscript presented in an intelligible fashion and written in standard English?

PLOS ONE does not copyedit accepted manuscripts, so the language in submitted articles must be clear, correct, and unambiguous. Any typographical or grammatical errors should be corrected at revision, so please note any specific errors here.

Reviewer #1: Yes

Reviewer #2: Yes

Reply: Thank you for your decision.

5. Review Comments to the Author

Please use the space provided to explain your answers to the questions above. You may also include additional comments for the author, including concerns about dual publication, research ethics, or publication ethics. (Please upload your review as an attachment if it exceeds 20,000 characters)

Reply: Thank you for your kind instruction.

6. PLOS authors have the option to publish the peer review history of their article ([what does this mean?](https://journals.plos.org/plosone/s/editorial-and-peer-review-process#loc-peer-review-history)). If published, this will include your full peer review and any attached files.


If you choose “no”, your identity will remain anonymous but your review may still be made public.


**Do you want your identity to be public for this peer review?** For information about this choice, including consent withdrawal, please see our [Privacy Policy](https://www.plos.org/privacy-policy).

Reviewer #1: No

Reviewer #2: No

[NOTE: If reviewer comments were submitted as an attachment file, they will be attached to this email and accessible via the submission site. Please log into your account, locate the manuscript record, and check for the action link "View Attachments". If this link does not appear, there are no attachment files.]

While revising your submission, please upload your figure files to the Preflight Analysis and Conversion Engine (PACE) digital diagnostic tool, <https://pacev2.apexcovantage.com/>. PACE helps ensure that figures meet PLOS requirements. To use PACE, you must first register as a user. Registration is free. Then, login and navigate to the UPLOAD tab, where you will find detailed instructions on how to use the tool. If you encounter any issues or have any questions when using PACE, please email PLOS at [figures@plos.org](http://hanmail/mail/MailComposeFrame.daum?TO=figures@plos.org). Please note that Supporting Information files do not need this step.

Reply: Thank you for your kind instruction.

Reviewer #1:

a. Question 1 → Although the work is very interesting and sound, the introduction is not completely linear and not adequately developed to soundly understand the work.

Reply: Thank you for your valuable comment. As per your comment, we have reviewed and revised the Introduction section as follows.

“**Introduction**

Standardized examination tools that reflect the sitting balance and psychological characteristics are scarce among survivors with significantly lowered balance and gait ability after stroke [1, 2]. In particular, it can be difficult to test the balance of stroke survivors using Likert ranking scales that examine sitting balance [1, 3]. The Berg Balance Scale (BBS) is used to investigate the sitting and standing balance [4], however, BBS may show a floor effect when examining stroke survivors who have decreased balance [5]. Additionally, the Functional Reaching Test, which examines the balance ability with multi-directional movement, is not seen as a meaningful balance examination. This is because scores may be affected by compensatory movement of the unaffected side of trunk [6].
Considering this limitation, Medley et al developed a form of Sitting Balance Scale (SBS) that can examine the patients who have decreased balance and gait abilities [1]. The SBS showed meaningful intra- and inter-rater reliability [1], it is valuable for sitting balance examination in older adults who are non-ambulatory or have limited functional mobility [2]. The SBS appears to be a valid, objective and comprehensive measure of a patient’s sitting balance ability. Also, Trunk Impairment Scale (TIS), Trunk Control Test (TCT), Postural Assessment Scale for Stroke-Trunk Control (PASS-TC) measure bed mobility, trunk control and sitting balance. The TIS and TCT have a relationship with the following tests: the gait subscale of Tinneti Performance Oriented Mobility Assessment (53%∼49%), Functional Ambulation Category (50%∼43%), 10-meter Walk Test (24%∼27%), TUG (36%∼44%) in stroke survivors [7]. The PASS-TC is correlated with BI (r=.89) and balance subscale of Fugl-Meyer Assessment (r=0.73) [8].

The normal gait requires a complex interaction of several factors, such as range of motion, muscle strength of lower extremities, nervous system, and trunk function. Timed Up & Go Test (TUG) is one of the most commonly used tools to test balance and mobility in clinical settings. Timed Up & Go test is correlated with gait performance and walking endurance in chronic stroke survivors [9].

As the results of these previous studies, sitting balance, trunk control and gait or mobility are correlated with each other, sitting balance [9-14]. However, it is not enough the evidence whether sitting balance and trunk control are related with mobility, and sitting balance and trunk control can predict the mobility level of sub-acute stroke survivors.
Therefore, the aim of this study was to investigate the correlation between sitting balance, trunk control, and mobility, and predictive validity for mobility in sub-acute stroke survivors.”

b. Question 1 → Please, at pag. 9 row 29-30, specify in a more detailed way the aim of the study.

Reply: Thank you for your comment. As per your comment, we have revised the aim of the study as follows.

“Therefore, the aim of this study was to investigate the correlation between sitting balance, trunk control, and mobility, and predictive validity for mobility in sub-acute stroke survivors.”

c. Question 4 → Even if the article is totally written in plain and sound English, at pag. 13 rows from 6 to 9, the specific phrase needs to be better explained.

Reply: Thank you for your detailed comment. The authors reviewed the sentences and made clearer. The changed contents are highlighted in red.

d. Please, at pag. 8 row 10, correct the initial “zero” number as written on all other decimal values in the list.

Reply: Thank you for your detailed comment. The “zero” number was added on all decimal values in the list.

e. Even if the statistical approach seems sound, at pag. 19, the two groups identified as: “Mobility available group” and “Mobility impaired group” seems to be too much different in population number (41 / 14).

Reply: Thank you for your valuable comment. We totally agree with your opinion. As per your comment, that point was added as one of limitations of this study in Discussion section as follows.

“This study have several limitations. First, the sample size in mobility impaired group was relatively small compared to number of mobility available group. In addition, the age group of the participants was relatively low. And the participants in TUG test were performed based on a specific point in time, thus, it may be limited in its adaptation to all stroke survivors. The second, Medley et al and Thompson reported that SBS is the most objective clinical examination method because it focuses on comprehensive balance examination rather than trunk control examination of TIS [16, 19]. Therefore, in SBS, which is traditionally used, it may be an appropriate examination tool to determine the discrimination of sitting balance and mobility in stroke patients with significantly impaired balance and gait performance. Future studies will need standardization to determine the superiority of sitting balance using convenience sampling of normal individuals and stroke patients. Lastly, the use of new examination tools will require research that reflects the psychological characteristics (sensitivity, specificity, response rate) of patients with various neurological disorders.”

f. Even if it has been declared that age is not a fundamental factor, it seems that all the subject population are young.

Reply: Thank you for your valuable comment. We totally agree with your opinion. As per your comment, that point was added as one of limitations of this study in Discussion section as follows.

“This study have several limitations. First, the sample size in mobility impaired group was relatively small compared to number of mobility available group. In addition, the age group of the participants was relatively low. And the participants in TUG test were performed based on a specific point in time, thus, it may be limited in its adaptation to all stroke survivors. The second, Medley et al and Thompson reported that SBS is the most objective clinical examination method because it focuses on comprehensive balance examination rather than trunk control examination of TIS [16, 19]. Therefore, in SBS, which is traditionally used, it may be an appropriate examination tool to determine the discrimination of sitting balance and mobility in stroke patients with significantly impaired balance and gait performance. Future studies will need standardization to determine the superiority of sitting balance using convenience sampling of normal individuals and stroke patients. Lastly, the use of new examination tools will require research that reflects the psychological characteristics (sensitivity, specificity, response rate) of patients with various neurological disorders.”

Reviewer #2: Review_PONE-D-20-25714

Major comments
#１. What is the significance of using trunk/sitting balance performance to predict mobility?
Please explain in the introduction. While trunk performance and/or sitting balance are important to mobility from previous research, I don’t think that as a reason to exclude other factors (e.g. lower extremity strength). What does reporting moderate predictive validity as a result of limited predictors lead to? Please also highlight the significance of this entire study in the introduction.

Reply: Thank you for your valuable comment. As per your comment, we have added some description about that and revised the Introduction section as follows.

“**Introduction** Standardized examination tools that reflect the sitting balance and psychological characteristics are scarce among survivors with significantly lowered balance and gait ability after stroke [1, 2]. In particular, it can be difficult to test the balance of stroke survivors using Likert ranking scales that examine sitting balance [1, 3]. The Berg Balance Scale (BBS) is used to investigate the sitting and standing balance [4], however, BBS may show a floor effect when examining stroke survivors who have decreased balance [5]. Additionally, the Functional Reaching Test, which examines the balance ability with multi-directional movement, is not seen as a meaningful balance examination. This is because scores may be affected by compensatory movement of the unaffected side of trunk [6].
Considering this limitation, Medley et al developed a form of Sitting Balance Scale (SBS) that can examine the patients who have decreased balance and gait abilities [1]. The SBS showed meaningful intra- and inter-rater reliability [1], it is valuable for sitting balance examination in older adults who are non-ambulatory or have limited functional mobility [2]. The SBS appears to be a valid, objective and comprehensive measure of a patient’s sitting balance ability. Also, Trunk Impairment Scale (TIS), Trunk Control Test (TCT), Postural Assessment Scale for Stroke-Trunk Control (PASS-TC) measure bed mobility, trunk control and sitting balance. The TIS and TCT have a relationship with the following tests: the gait subscale of Tinneti Performance Oriented Mobility Assessment (53%∼49%), Functional Ambulation Category (50%∼43%), 10-meter Walk Test (24%∼27%), TUG (36%∼44%) in stroke survivors [7]. The PASS-TC is correlated with BI (r=.89) and balance subscale of Fugl-Meyer Assessment (r=0.73) [8].

The normal gait requires a complex interaction of several factors, such as range of motion, muscle strength of lower extremities, nervous system, and trunk function. Timed Up & Go Test (TUG) is one of the most commonly used tools to test balance and mobility in clinical settings. Timed Up & Go test is correlated with gait performance and walking endurance in chronic stroke survivors [9].

As the results of these previous studies, sitting balance, trunk control and gait or mobility are correlated with each other, sitting balance [9-14]. However, it is not enough the evidence whether sitting balance and trunk control are related with mobility, and sitting balance and trunk control can predict the mobility level of sub-acute stroke survivors.
Therefore, the aim of this study was to investigate the correlation between sitting balance, trunk control, and mobility, and predictive validity for mobility in sub-acute stroke survivors.”

#2. Most importantly, I don’t see the clinical significance of predicting current TUG in a patient with measurable TUG. What is important is not predicting TUG, but that the mobility to be assessed by TUG can be explained a lot by sitting balance?

Reply: Thank you for your valuable comment. Through this study, we have tried to investigate mainly whether sitting balance and trunk control can have predictive validity for predicting mobility level by TUG. So, we have added description about correlation between TUG and gait performance in chronic stroke patients in Introduction section, as well as another descriptions, as above.

3. The two objectives seem to create confusion. What you want to clarify is the correlation between TUG and other performance test? Or is it trunk/sitting performance as a predictor of TUG? The conclusions do not seem to emphasize the correlation.

Reply: Thank you for your valuable comment. As per your comment, we have revised the aim of the study as follows.

“Therefore, the aim of this study was to investigate the correlation between sitting balance, trunk control, and mobility, and predictive validity for mobility in sub-acute stroke survivors.”

4. Although your study is a cross-sectional design, there are several representations that can be used to predict future mobility. Please give appropriate consideration to the information available from the results.

Reply: Thank you for your valuable comment. As per your comment, for demostrating about that, we have revised the Results section and Discussion section as follows.

“**Results**
There were no significant differences in age, sex, disease duration, etiology type, affected sides between mobility available group and mobility impaired group, however, in TUG, SBS, TCT, PASS-TC, TIS, there were significant differences between two groups (Table 1).

TUG is significantly correlated with SBS (r = -0.78), TIS (r = -0.76), TCT (r = -0.65), and PASS-TC (r = -0.67) (Table 2).

The ROC curve analysis showed that the SBS cut-off value was > 28.5, TIS > 16.5, TCT > 82, and PASS-TC > 10.5. All four examinations showed that the AUC was a tool for moderate accuracy (0.84 to 0.90, *p* < 0.001) (Table 3).

SBS (β = -0. 337) and TIS (β = -0. 317) were found to have a 65% effect on the TUG (Table 4). The result from the logistic regression analysis predicted the mobility by using the cut-off value of each examination.

An ROC curve analysis, revealed that SBS (> 28.5 score, odds ratio 0.62) was the most influential variable (Table 5).

**Discussion**
The sitting balance and trunk control after stroke can be impaired, and to solve these problems is important for successful rehabilitation of stroke survivors [13, 14]. In particular, impaired sitting balance and trunk control are occurred by hemiplegic side of trunk [23], these factors can limit the functional activities of stroke survivors. The results of the present study demonstrated that mobility impaired group had significantly decreased mobility (TUG), sitting postural balance (SBS), and trunk control (TIS, TCT, PASS-TC) than mobility available group. The mobility by TUG was found to be significantly related to sitting balance (SBS) and trunk control (TIS, TCT, PASS-TC). The present study, cut-off values of SBS, TCT, PASS-TC and TIS were used as a reference point for estimating the mobility level. In these examinations, it was confirmed that the AUC was moderately accurate (curve area = 0.84 ~ 0.90) [22]. In addition, for the first time, we presented a selection criterion for predicting mobility level. Specificity was acceptable in all four examinations (78% to 87%), specificity (71% to 85%) and positive predictive value (90% to 94%), negative predictive value (57% ~ 68%) was somewhat low.
Through the present study, the SBS demonstrated the highest correlation for mobility using TUG. Also, SBS was revealed as the most dominant examination tool predicting the mobility. It can be explained that the SBS contains the postural balance compared to the other trunk control tests, postural balance can be a meaningful predictor the improvement of mobility in sub-acute stroke survivors. As a result of this study, prediction of mobility level for stroke survivors who have 4 months of disease duration and minimal to moderate paralysis was found that SBS, TIS, TCT, and PASS-TC are excellent screening methods and show positive predictive value because of sensitivity, specificity and screening test. The negative predictive value of these tests was found to be somewhat low. As in previous studies [2, 15], the study classified that if the time to perform a TUG test is greater than 20 seconds, It’s a mobility available group that can walk and if the time to perform a TUG test is equal or less than 20 seconds, it's a mobility impaired group that can’t walk. Specifically, the stroke survivors who needed considerably short time for examining TUG, have inappropriate compensatory strategy and use assistive device but independent mobility is possible may not be included. Therefore, more survivors would be required and a more quantitative approach is needed for the classification criteria of mobility level. However, in this study, the selection criteria for discriminating the mobility level in the sitting balance (SBS) and trunk control (TIS, TCT, and PASS-TC) is that sitting balance and trunk control should be maintained before functional movement and restoration of the preceding postural control and mobility.
TCT and PASS-TC are suitable for use in acute and subacute patients. The TCT and PASS-TC scores of the mobility impaired group in this study were 74 (100 out of 100) and 9.5 (15 out of 12), respectively, but SBS (21.36 points / 40 points) and TIS (11.93 points / 23 points) of mobility impaired group were significantly lower than mobility available group. In addition, the sitting balance and trunk control, capable of moving the predetermined section on the TUG test contents, are necessary components because SBS and TIS examination items are also large part of the tasks required to perform the TUG.
The relationship between SBS and TIS for the elderly was also verified in various conditions (acute phase patient nursing r = 0.92, rehabilitation nursing r = 0.89, intensive nursing r = 0.88, home nursing r = 0.60); both variables are very closely related [2]. In contrast, TCT and PASS-TC consist of only four to five items that examine mobility in bed. Since there is no item that can affect the TUG, it can be seen as a low examination of discrimination tool. In particular, TCT is not capable of qualitative examination of trunk movement [19], and there was a moderate correlation with the trunk muscle strength using the dynamometer [24].

In the present study, it was confirmed that SBS has the highest predictive validity in discriminating mobility level. Unlike TIS, TCT, and PASS-TC, the SBS is consisted of controlling the upper and lower trunk and coordination, as well as a specific task item for examining the comprehensive dynamic balance capability, required for sit to stand task and mobility. This was also confirmed in the factor analysis (except for TCT and PASS-TC) affecting TUG. In the Rasch analysis, static section among TIS items was not suitable because of the ceiling effect in subacute and chronic stroke survivors, this is also seemed to have not affected the power of discrimination of static section [25, 26].
This study have several limitations. First, the sample size in mobility impaired group was relatively small compared to number of mobility available group. In addition, the age group of the participants was relatively low. And the participants in TUG test were performed based on a specific point in time, thus, it may be limited in its adaptation to all stroke survivors. The second, Medley et al and Thompson reported that SBS is the most objective clinical examination method because it focuses on comprehensive balance examination rather than trunk control examination of TIS [16, 19]. Therefore, in SBS, which is traditionally used, it may be an appropriate examination tool to determine the discrimination of sitting balance and mobility in stroke patients with significantly impaired balance and gait performance. Future studies will need standardization to determine the superiority of sitting balance using convenience sampling of normal individuals and stroke patients. Lastly, the use of new examination tools will require research that reflects the psychological characteristics (sensitivity, specificity, response rate) of patients with various neurological disorders.”

5. What is the significance or hypothesis of the MBI that was evaluated? Do you just want to investigate the correlation to TUG? ADLs were discussed in the previous studies presented in the introduction, but they were not mentioned in the DISCUSSION.

Reply: Thank you for your valuable comment. MBI was not main parameter in the study. So, as per your comment, we have deleted related description about MBI.

Minor comments

overall
#1. In the title, aim, and abstract, you use “among sitting balance, trunk control, mobility, and activities daily living" and "between sitting balance, trunk control, mobility and ADL”, it's hard to understand what you want to compare them to.
The results only show an correlation between mobility and other factors.
This can lead to a misunderstanding and a misguided purpose, as if it investigated a correlation among all factors.

Reply: Thank you for your valuable comment. As per your comment, we have revised the title, aim, and abstract of the study as follows.

**“Title: Correlation between mobility and sitting postural control, and predictive validity of for mobility level in survivors with sub-acute stroke”**

**“ABSTRACT**

**Purpose:** To investigate the correlation between sitting balance, trunk control, and mobility, as well as to predict the mobility level in sub-acute stroke survivors.

**Design:** A observational and cross-sectional study.

**Methods:** Sixty-five hemiplegic stroke survivors were participated in this study. The Timed Up and Go Test (TUG) was used to estimate mobility, and the Sitting Balance Scale (SBS) was used to examining sitting balance. The Trunk Impairment Scale (TIS), Trunk Control Test (TCT), and Postural Assessment Scale for Stroke-trunk control (PASS-TC) were used for examining the trunk control.

**Findings:** The TUG is significantly correlated with SBS (r = -0.78), TIS (r = -0.76), TCT (r = -0.65), and PASS-TC (r = -0.67). In addition, the receiver operation characteristic (ROC) curve showed as cut-off value of SBS as >28.5, TIS > 16.5, TCT >82, and PASS-TC >10.5. The area under the ROC curve in each of the four tests is moderately accurate for predicting the mobility of sub-acute stroke survivors (.84 ~ .90).

**Conclusion:** The SBS showed the highest correlation for mobility using TUG in sub-acute stroke survivors. Also, SBS was revealed as the most dominant examination tool predicting the mobility by TUG, it can be explained the sitting postural balance is the variable predicting the mobility in sub-acute stroke survivors.**”**

**“**Therefore, the aim of this study was to investigate the correlation between sitting balance, trunk control, and mobility, and predictive validity for mobility in sub-acute stroke survivors.**”**

#2. The key word was “chronic stroke”, is that correct?

Reply: Thank you for your detailed comment. One of the key word of this study is sub-acute stroke, the “chronic stroke” is incorrect. So, we have revised the expression.

abstract
#3. P2, L2. Is it gait ability or mobility that you predict? If you don't distinguish and define it, then please unify.

Reply: Thank you for your detailed comment. As per your comment, we have deleted the expression ‘gait’ in the Abstract section.

#4. P2, L10. Is the Finding notation TCT (r = -0.65)? There should be a space before and after “=”.

Reply: Thank you for your detailed comment. The authors corrected the notations for insert a space before and after “=”.

#5. P2, L11-14. Please indicate what the ROC curve or AUC is intended to do. to predict mobility?

Reply: Thank you for your detailed comment. As per your comment, we have added the description about that in Abstract as follows.
“In addition, the receiver operation characteristic (ROC) curve showed as cut-off value of SBS as >28.5, TIS > 16.5, TCT >82, and PASS-TC >10.5. The area under the ROC curve in each of the four tests is moderately accurate for predicting the mobility of sub-acute stroke survivors (.84 ~ .90).”

#6. P2, L17. “...predicting the improvement of mobility after stroke.” does not prove improvement from this cross-sectional design.

Reply: Thank you for your detailed comment. The design of the present study is cross-sectional design, so, we deleted the word “improvement” to clarify the conclusion.

Introduction
#7. P3, L10-30. The introduction as a whole hypothesizes the usefulness of SBS and these indicators as tools that can predict ADLs. Nevertheless, as a purpose, SBS is not the subject matter and what it predicts is not ADL. So, It’s hard to understand the flow of why you want to predict mobility. The connection from background to purpose seems somewhat forced. Please clarify the hypothesis and revise the structure.

Reply: Thank you for your valuable comment. We have revised some description in Introduction section to clarify the aim of this study as follows.

“**Introduction**
Standardized examination tools that reflect the sitting balance and psychological characteristics are scarce among survivors with significantly lowered balance and gait ability after stroke [1, 2]. In particular, it can be difficult to test the balance of stroke survivors using Likert ranking scales that examine sitting balance [1, 3]. The Berg Balance Scale (BBS) is used to investigate the sitting and standing balance [4], however, BBS may show a floor effect when examining stroke survivors who have decreased balance [5]. Additionally, the Functional Reaching Test, which examines the balance ability with multi-directional movement, is not seen as a meaningful balance examination. This is because scores may be affected by compensatory movement of the unaffected side of trunk [6].
Considering this limitation, Medley et al developed a form of Sitting Balance Scale (SBS) that can examine the patients who have decreased balance and gait abilities [1]. The SBS showed meaningful intra- and inter-rater reliability [1], it is valuable for sitting balance examination in older adults who are non-ambulatory or have limited functional mobility [2]. The SBS appears to be a valid, objective and comprehensive measure of a patient’s sitting balance ability. Also, Trunk Impairment Scale (TIS), Trunk Control Test (TCT), Postural Assessment Scale for Stroke-Trunk Control (PASS-TC) measure bed mobility, trunk control and sitting balance. The TIS and TCT have a relationship with the following tests: the gait subscale of Tinneti Performance Oriented Mobility Assessment (53%∼49%), Functional Ambulation Category (50%∼43%), 10-meter Walk Test (24%∼27%), TUG (36%∼44%) in stroke survivors [7]. The PASS-TC is correlated with BI (r=.89) and balance subscale of Fugl-Meyer Assessment (r=0.73) [8].

The normal gait requires a complex interaction of several factors, such as range of motion, muscle strength of lower extremities, nervous system, and trunk function. Timed Up & Go Test (TUG) is one of the most commonly used tools to test balance and mobility in clinical settings. Timed Up & Go test is correlated with gait performance and walking endurance in chronic stroke survivors [9].

As the results of these previous studies, sitting balance, trunk control and gait or mobility are correlated with each other, sitting balance [9-14]. However, it is not enough the evidence whether sitting balance and trunk control are related with mobility, and sitting balance and trunk control can predict the mobility level of sub-acute stroke survivors.
Therefore, the aim of this study was to investigate the correlation between sitting balance, trunk control, and mobility, and predictive validity for mobility in sub-acute stroke survivors.”

#8. P2, L1-5. The use of the sitting balance tools scarce and BBS is explained at the beginning of the Introduction, but in the context of the “overall, SBS, Trunk Impairment Scale…(P3, L14-16)” there are other assessments that can be measured in the supine and sitting positions besides the SBS, Please explain these discrepancies.

Reply: Thank you for your detailed comment. As per your comment, we have revised the Introduction section as above.

#9. With regard to trunk control and sitting balance used in various contexts, it is often difficult to separate them. For example, the TCT and TIS also include sitting balance as an entry. Are there any definitions used in this study and any differences between there two elements?

Reply: Thank you for your detailed comment. We have tried to describe about the two words (sitting balance and trunk control) throughout the text as follows.

**“Title: Correlation between mobility and sitting postural control, and predictive validity of for mobility level in survivors with sub-acute stroke”**

**“Introduction**
Standardized examination tools that reflect the sitting balance and psychological characteristics are scarce among survivors with significantly lowered balance and gait ability after stroke [1, 2]. In particular, it can be difficult to test the balance of stroke survivors using Likert ranking scales that examine sitting balance [1, 3]. The Berg Balance Scale (BBS) is used to investigate the sitting and standing balance [4], however, BBS may show a floor effect when examining stroke survivors who have decreased balance [5]. Additionally, the Functional Reaching Test, which examines the balance ability with multi-directional movement, is not seen as a meaningful balance examination. This is because scores may be affected by compensatory movement of the unaffected side of trunk [6].
Considering this limitation, Medley et al developed a form of Sitting Balance Scale (SBS) that can examine the patients who have decreased balance and gait abilities [1]. The SBS showed meaningful intra- and inter-rater reliability [1], it is valuable for sitting balance examination in older adults who are non-ambulatory or have limited functional mobility [2]. The SBS appears to be a valid, objective and comprehensive measure of a patient’s sitting balance ability. Also, Trunk Impairment Scale (TIS), Trunk Control Test (TCT), Postural Assessment Scale for Stroke-Trunk Control (PASS-TC) measure bed mobility, trunk control and sitting balance. The TIS and TCT have a relationship with the following tests: the gait subscale of Tinneti Performance Oriented Mobility Assessment (53%∼49%), Functional Ambulation Category (50%∼43%), 10-meter Walk Test (24%∼27%), TUG (36%∼44%) in stroke survivors [7]. The PASS-TC is correlated with BI (r=.89) and balance subscale of Fugl-Meyer Assessment (r=0.73) [8].

The normal gait requires a complex interaction of several factors, such as range of motion, muscle strength of lower extremities, nervous system, and trunk function. Timed Up & Go Test (TUG) is one of the most commonly used tools to test balance and mobility in clinical settings. Timed Up & Go test is correlated with gait performance and walking endurance in chronic stroke survivors [9].

As the results of these previous studies, sitting balance, trunk control and gait or mobility are correlated with each other, sitting balance [9-14]. However, it is not enough the evidence whether sitting balance and trunk control are related with mobility, and sitting balance and trunk control can predict the mobility level of sub-acute stroke survivors.
Therefore, the aim of this study was to investigate the correlation between sitting balance, trunk control, and mobility, and predictive validity for mobility in sub-acute stroke survivors.**”**

Methods
#10. P4, L1-2. There is no mention of walking ability in the subject recruitment, is there a standard? At this time of year, I don't think everyone is capable of TUG. Or should readers understand that only those who are able to walk on advertisement have applied?

Reply: Thank you for your detailed comment. As per your comment, we have added inclusion criteria as follows.
“Inclusion criteria were as follows: who have Mini Mental Status Examination > 24, and who are able to walk 5meters with – or without assistive device.”

#11. P4, L3-4. Related to the above, what are the reasons for drop-outs in cross-sectional studies? Does this mean that there was a request from the patient, but it took a long time for the actual measurement to be taken, resulting in health deterioration and discharge?

Reply: Thank you for your detailed comment. In fact, the experiment was conducted a few days after the recruitment. In the meantime, applicants who were in poor health or discharged from the hospital were eliminated from the study. That's what it means.

#12. P4, L2. What is your rationale for using MMSE > 23 as the inclusion criteria? Does it appear that cognitive ability has a significant impact on the content of this assessment?

Reply: Thank you for your detailed comment. As per your comment, we have revised the description and added a reference.

“

#13. P4, L13. Can you indicate the extent of “...experience in using these tools”? It seems ambiguous.

Reply: Thank you for your comment. This sentence seems ambiguous as your advice, we revised the sentence clearer. The changed contents are highlighted in red.

#14. P4, L20-24. Please indicate the original citation for the TUG at the beginning of the description of the TUG (Reference No. 14). In addition, please describe the speed conditions of the TUG.

Reply: Thank you for your comment. The citation for the TUG corrected in first sentence of the paragraph. The changed contents are highlighted in red.

#15. P4, L28-30. Please reiterate the reference of SBS reliability.

Reply: Thank you for your comment. The reference for the SBS reliability was added in the paragraph. The changed contents are highlighted in red.

#16. P5, L12-13. Please reiterate the reference of TCT validity.

Reply: Thank you for your comment. The reference for the TCT validity was added in the paragraph. The changed contents are highlighted in red.

#17. P5, 34-35. What was the reason for comparing the two groups using only the Chi-square test after confirming the normal distribution in the statistical analysis?

Reply: Thank you for your valuable comment. As per your comment, we have analyzed the nominal data using the Chi-square test, and other data were analyzed by Mann-Whitney U test. We have revised the description as follows.

“Chi square test and Man-Whitney U test was used to compare the general characteristics between mobility available group and mobility impaired group.”

Results
#18. P6, L20. SBS and TIS have similar numbers in the results. What are the correlation coefficients between these two variables?　Discussion also states that it is very closely related [2]. Even though the concepts are different, we get the impression that the content of the test is also partially common. It is recommended to emphasize the concept and relationship between the two variables in the introduction or discussion.

Reply: Thank you for your valuable comment. As per your comment, we have revised the Introduction section and Discussion section.

Discussion
#19. P6, L29-30. “The results of the present study...had significantly reduced mobility (TUG),” is natural because it generates two groups according to TUG. It is an unnecessary expression.

Reply: Thank you for your comment. As per tour comment, we have reviewed and revised the Discussion section as follows.

**“Discussion**
The sitting balance and trunk control after stroke can be impaired, and to solve these problems is important for successful rehabilitation of stroke survivors [13, 14]. In particular, impaired sitting balance and trunk control are occurred by hemiplegic side of trunk [23], these factors can limit the functional activities of stroke survivors. The results of the present study demonstrated that mobility impaired group had significantly decreased mobility (TUG), sitting postural balance (SBS), and trunk control (TIS, TCT, PASS-TC) than mobility available group. The mobility by TUG was found to be significantly related to sitting balance (SBS) and trunk control (TIS, TCT, PASS-TC). The present study, cut-off values of SBS, TCT, PASS-TC and TIS were used as a reference point for estimating the mobility level. In these examinations, it was confirmed that the AUC was moderately accurate (curve area = 0.84 ~ 0.90) [22]. In addition, for the first time, we presented a selection criterion for predicting mobility level. Specificity was acceptable in all four examinations (78% to 87%), specificity (71% to 85%) and positive predictive value (90% to 94%), negative predictive value (57% ~ 68%) was somewhat low.
Through the present study, the SBS demonstrated the highest correlation for mobility using TUG. Also, SBS was revealed as the most dominant examination tool predicting the mobility. It can be explained that the SBS contains the postural balance compared to the other trunk control tests, postural balance can be a meaningful predictor the improvement of mobility in sub-acute stroke survivors. As a result of this study, prediction of mobility level for stroke survivors who have 4 months of disease duration and minimal to moderate paralysis was found that SBS, TIS, TCT, and PASS-TC are excellent screening methods and show positive predictive value because of sensitivity, specificity and screening test. The negative predictive value of these tests was found to be somewhat low. As in previous studies [2, 15], the study classified that if the time to perform a TUG test is greater than 20 seconds, It’s a mobility available group that can walk and if the time to perform a TUG test is equal or less than 20 seconds, it's a mobility impaired group that can’t walk. Specifically, the stroke survivors who needed considerably short time for examining TUG, have inappropriate compensatory strategy and use assistive device but independent mobility is possible may not be included. Therefore, more survivors would be required and a more quantitative approach is needed for the classification criteria of mobility level. However, in this study, the selection criteria for discriminating the mobility level in the sitting balance (SBS) and trunk control (TIS, TCT, and PASS-TC) is that sitting balance and trunk control should be maintained before functional movement and restoration of the preceding postural control and mobility.
TCT and PASS-TC are suitable for use in acute and subacute patients. The TCT and PASS-TC scores of the mobility impaired group in this study were 74 (100 out of 100) and 9.5 (15 out of 12), respectively, but SBS (21.36 points / 40 points) and TIS (11.93 points / 23 points) of mobility impaired group were significantly lower than mobility available group. In addition, the sitting balance and trunk control, capable of moving the predetermined section on the TUG test contents, are necessary components because SBS and TIS examination items are also large part of the tasks required to perform the TUG.
The relationship between SBS and TIS for the elderly was also verified in various conditions (acute phase patient nursing r = 0.92, rehabilitation nursing r = 0.89, intensive nursing r = 0.88, home nursing r = 0.60); both variables are very closely related [2]. In contrast, TCT and PASS-TC consist of only four to five items that examine mobility in bed. Since there is no item that can affect the TUG, it can be seen as a low examination of discrimination tool. In particular, TCT is not capable of qualitative examination of trunk movement [19], and there was a moderate correlation with the trunk muscle strength using the dynamometer [24].

In the present study, it was confirmed that SBS has the highest predictive validity in discriminating mobility level. Unlike TIS, TCT, and PASS-TC, the SBS is consisted of controlling the upper and lower trunk and coordination, as well as a specific task item for examining the comprehensive dynamic balance capability, required for sit to stand task and mobility. This was also confirmed in the factor analysis (except for TCT and PASS-TC) affecting TUG. In the Rasch analysis, static section among TIS items was not suitable because of the ceiling effect in subacute and chronic stroke survivors, this is also seemed to have not affected the power of discrimination of static section [25, 26].
This study have several limitations. First, the sample size in mobility impaired group was relatively small compared to number of mobility available group. In addition, the age group of the participants was relatively low. And the participants in TUG test were performed based on a specific point in time, thus, it may be limited in its adaptation to all stroke survivors. The second, Medley et al and Thompson reported that SBS is the most objective clinical examination method because it focuses on comprehensive balance examination rather than trunk control examination of TIS [16, 19]. Therefore, in SBS, which is traditionally used, it may be an appropriate examination tool to determine the discrimination of sitting balance and mobility in stroke patients with significantly impaired balance and gait performance. Future studies will need standardization to determine the superiority of sitting balance using convenience sampling of normal individuals and stroke patients. Lastly, the use of new examination tools will require research that reflects the psychological characteristics (sensitivity, specificity, response rate) of patients with various neurological disorders.**”**

#20. P6, L29-34. Several repetitions of the results are described. Please summarize (especially the numbers) and organize them.

Reply: Thank you for your comment. As per your comment, we have reviewed and revised the Discussion section as above.

#21. P7, L11-13. The sentence of “Specifically, the stroke suvivors ... may not be included.”, what is your rationale for describing the inappropriate compensatory strategy and use assistive device? The authors don't consider the assistive device in recruitment and allow it to be used in the TUG, but what was the device usage rate?

Reply: Thank you for your comment. As per your comment, we have reviewed and revised the Discussion section as above.

#22. P7, L15-18. What do the authors mean by “However, in this study,... postural control and mobility." It is somewhat difficult to understand the implications of these sentences.
Is there any evidence to be drawn from the results of this context or from previous studies?”

Reply: Thank you for your comment. As per your comment, we have added the reference for the description.

#23. P7, L19-23. The sentence of “The TCT and ... for TIS, respectively)”, this is the kind of sentence is a repetition of the results, or it should be written in the Results section.

Reply: Thank you for your comment. As per tour comment, we have reviewed and revised the Discussion section as above.

#24. P7, L19. Please state the rationales or add a citation for “the TCT and PASS-TC are suitable for use in acute and subacute patients.”

Reply: Thank you for your comment. As per your comment, we have reviewed and revised the Discussion section as above.

#25. P7, L23-25. There is a lack of textual basis or citation for “In addition, the balance…required to perform the TUG.”

Reply: Thank you for your kind comments. As per your comment, we have reviewed and revised the Discussion section as above.

#26. P8, L4-6. What do these citations explain about “Medley et al and …examination of TIS [15, 18]”? Since SBS and TIS are newer reports than these articles, please clarify what you mean by these citations. Also, what do you mean by “in SBS, which is traditionally used,” in the sentence that follows?

Reply: Thank you for your kind comments. As per your comment, we have reviewed and revised the Discussion section as above.

#27. Please make the Limitation clear. As I see it, there are several limitations.

Reply: Thank you for your valuable comment. As per your comment, at the end of the discussion, the limitations of this study were described in order as follows.

“This study have several limitations. First, the sample size in mobility impaired group was relatively small compared to number of mobility available group. In addition, the age group of the participants was relatively low. And the participants in TUG test were performed based on a specific point in time, thus, it may be limited in its adaptation to all stroke survivors. The second, Medley et al and Thompson reported that SBS is the most objective clinical examination method because it focuses on comprehensive balance examination rather than trunk control examination of TIS [16, 19]. Therefore, in SBS, which is traditionally used, it may be an appropriate examination tool to determine the discrimination of sitting balance and mobility in stroke patients with significantly impaired balance and gait performance. Future studies will need standardization to determine the superiority of sitting balance using convenience sampling of normal individuals and stroke patients. Lastly, the use of new examination tools will require research that reflects the psychological characteristics (sensitivity, specificity, response rate) of patients with various neurological disorders.”

Table 1.
#28. What is the meaning of †?

Reply: Thank you for your detailed comment. As per your comment, we have deleted that.

#29. The percentages of the paralysis location are not shown.

Reply: Thank you for your comment. As per your comment, we have added the percentage s of the paralysis location in the Table 1.

Table 3.
#30. * does not appear to be in the table. Please remove it.

Reply: Thank you for your comment. As per your comment, we have deleted that.

#31. Is this table showing a cut-off score to predict the mobility available group?

Reply: Thank you for your valuable comment. As per your comment, we have deleted that. The receiver operation characteristic (ROC) curve showed as cut-off value of SBS as >28.5, TIS > 16.5, TCT >82, and PASS-TC >10.5. The area under the ROC curve in each of the four tests is moderately accurate for predicting the mobility of sub-acute stroke survivors (.84 ~ .90). It means that if SBS >28.5 points, the probability of predicting or distinguishing the person who can walk is 80% (sensitivity), and if SBS <28.5 points, the possibility of predicting or distinguishing the person who cannot walk is 78%(specificity)
