# Supplementary material for: The relationship between sitting balance, trunk control and mobility with predictive for current mobility level in survivors of sub-acute stroke
Source: PLoS One. 2021 Aug 5;16(8):e0251977. doi: 10.1371/journal.pone.0251977 (PMC8341576; doi:10.1371/journal.pone.0251977)
Supplement: Attachment — Submitted filename: review comments_revised.docx [file pone.0251977.s003.docx]

I have checked your revised manuscript, but there are still some things that you cannot responded. Please refer to the following comments to improve your manuscript.

**Major comments**

#1. What is the significance of using trunk/sitting balance performance to predict mobility? The significance/necessity of this study has not been demonstrated.

The introduction is still not clear and linear. Please clarify the following:

・What is the significance of targeting the sub-acute phase?

・The purpose is not clear. Make it clear whether you want to see relationships or predict mobility. The latter seems to be written like a main message, but if so, it needs to be explicit in the introduction (TUG is the subject in the Findings).

・Please state your research hypothesis. If you want to reveal scale properties in an exploratory manner, please insist on doing so. I don’t understand why mobility is suddenly mentioned at the end of Introduction.

#2. Again, you will also need to answer the following question.

“Most importantly, I don’t see the clinical significance of predicting current TUG in a patient with measurable TUG. What is important is not predicting TUG, but that the mobility to be assessed by TUG can be explained a lot by sitting balance?”

Previous studies have proposed the importance of trunk/sitting balance performance to predict future TUG (not the current TUG).

#3. Related to the above, the overall structure of the introduction section could be further improved. The introduction flow sounds like it emphasizes the advantage of SBS, but is doesn’t seem to be connected to the hypothesis or purpose. Isn’t the flow that SBS is supposed to reflect TUG more than TCT, TIS, or PASS-TC?

If you want to emphasize SBS, the significance of the description of BBS and FRT before it appears to be unimportant. Is SBS an alternative to BBS and FRT? It might be more appropriate to emphasize mobility first instead.

For example.

1. General remarks: stroke and their balance and gait ability

2. Mobility: the significance and necessity of predicting mobility

3. Current status of indices proposed for this purpose

4. Validity of the SBS

5. Hypothesis and purpose

In particular, “2. Mobility“ is important because they determine the significance of this study.

Again, is there a need to bother predicting the current TUG?

If you change the introduction, please reorganize the discussion with the corresponding text.

**Minor comments**

#4. Title

It may need to be modified to meet the purpose of the text.

Consider whether it is appropriate to use the term "predictive" for current mobility.

For example, it might be as simple as "the relationship between trunk/sitting balance performance and mobility".

#5. Abstract

Your purpose is the same as the title. Isn't the main message to identify what can predict mobility level?　What is the significance of looking at the relationship between clinical indicators?

Is there a specific reason to focus on the sub-acute phase?　If not, this information should be in Methods.

Methods:

The sample size of 65 participants in methods is misleading, and the actual number of participants (55) needs to be indicated. There is no information at all to process the predictions. It is just a list of scales, but it is not a research method.

Findings:

Isn't ROC to “predict the TUG”? Results about predictions are not describe in the methods.

Please standardize the notation of 0 ( .84 or -0.78).

Conclusion:

“Sub-acute stroke survivors” is repeated in the Conclusion. Other redundant expressions are also noticeable

#6. Introduction

You describe the relationship between each indicator, but I don't understand what you are trying to explain. Is the purpose of your research to verify the validity of the scale? It is not appropriate to say that you examined the relationship because it is unknown. Please discuss what you are trying to say by examining the relationship.

#7. Introduction

What does the percentages in the relationship sentences in TIS and TCT stand for?

#8. Introduction

Please do not abbreviate the BI of the first appearance.

#9. Discussion

The discussion is redundant (e.g., repetition of the same terms, repetition of the same kind of arguments). Please organize your arguments.

#10. Discussion

Regarding the sentence "For the first time, we presented a selection criterion for predicting mobility level", as pointed out in the intro, I did not understand the need to show the predicted TUG for those who are capable of TUG using other indicators. Please include this reason and emphasize it again in your discussion. Otherwise, the significance of the study shown “for the first time” will not be conveyed.

#11. Discussion

“It's a mobility available group that can walk and if the time to perform a TUG test is equal or less than 20 seconds, it's a mobility impaired group that can't walk”.

What is the point you are trying to make with these related sentences? Is it a repetition of the methodology?

#12. Conclusion

The conclusion does not receive your purpose. The objective to lead this conclusion is “does the sitting balance correlate with current mobility?” I'm not sure if “prediction” is the right term, but it is common for linear prediction to hold if there is a correlation.

Isn't the basic argument and conclusion not the validity of the predictions, but how the sitting balance relates to mobility?

If you want to argue for predictive validity, I think the title and purpose should reflect “the validity of the sitting balance that predicts the current TUG”.

If the latter, sitting balance is highly correlated with mobility, so it is important to focus on sitting balance in sub-acute stroke.

Additional comment

There are sentences that need to be consistent with the time period as a whole. In particular, the report of previous studies should be shown in the past tense.
