# Supplementary material for: The relationship between sitting balance, trunk control and mobility with predictive for current mobility level in survivors of sub-acute stroke
Source: PLoS One. 2021 Aug 5;16(8):e0251977. doi: 10.1371/journal.pone.0251977 (PMC8341576; doi:10.1371/journal.pone.0251977)
Supplement: Attachment — Submitted filename: Response to comments.docx [file pone.0251977.s004.docx]

**Response to comments**

**Major comments**

#1. What is the significance of using trunk/sitting balance performance to predict mobility? The significance/necessity of this study has not been demonstrated.

The introduction is still not clear and linear. Please clarify the following:

1) What is the significance of targeting the sub-acute phase?

Answer) Thank you for your question. Generally, most stroke patients in acute stage may be still medically unstable and have not started rehabilitation in earnest. And, in chronic stroke patients, recovery was almost advanced, and the recovery rate was slow or nearly reached a plateau. Thus, we investigated for sub-acute stroke patients. Subsequently we tried to verify the relationship between subject's sitting balance and trunk control and mobility, and predictive validity of the sitting balance and trunk control for predicting mobility level.

2) The purpose is not clear. Make it clear whether you want to see relationships or predict mobility. The latter seems to be written like a main message, but if so, it needs to be explicit in the introduction (TUG is the subject in the Findings).

Answer) Thank you for your kind comments. According to your advice, we have rewritten the Introduction section to make it clearer that the order and content of manuscripts have been changed to further emphasize the meaning of SBS. All of changed contents are highlighted in red.

**“Introduction**
Hemiparesis of stroke survivors can reduce the function of trunk and extremities, resulting in impaired sitting and standing balance. Decreased balance ability is a common symptom due to stroke, it can affects gait and activities of daily living [1]. Hence, the balance evaluation of stroke patients is one of the essential factors that can assess the functional level of the stroke patients.

Balance can be measured in a sitting and standing position [2]. Most patients with acute and subacute stroke have a poor sitting balance, they cannot maintain the standing posture. Since, prediction of mobility after stroke is possible to only a few of these patients. However, the mobility is needed for independent daily living, goal setting of rehabilitation through mobility prediction is necessary for all stroke patients. One of the physical goal of stroke rehabilitation is to restore the level of mobility, it is imperative to inform patients and their family of possible levels of mobility recovery to lead a normal social life. In addition, it is needed that prediction of the mobility level can induce active participation in rehabilitation through noticeable motivation. Since, previous studies investigated the prediction for mobility by evaluating post-stroke balance dysfunction. [3-5].
The Berg Balance Scale (BBS) is used to investigate the sitting and standing balance [6], however, BBS may show a floor effect when examining stroke survivors who have decreased balance [7]. In particular, it can be difficult to test the balance of stroke survivors using Likert ranking scales that examine sitting balance [8, 9].

Considering this limitation, Medley et al developed a form of Sitting Balance Scale (SBS) that can examine the patients who have decreased balance and gait abilities [8]. The SBS showed meaningful intra- and inter-rater reliability [8], it is valuable for sitting balance examination in older adults who are non-ambulatory or have limited functional mobility [10]. The SBS appears to be a valid, objective and comprehensive measure of a patient’s sitting balance ability.

There are other tools for assessing sitting balance, such as the Trunk Impairment Scale (TIS), Trunk Control Test (TCT), Postural Assessment Scale for Stroke-Trunk Control (PASS-TC). The evaluation tools measure bed mobility, trunk control and sitting balance. The TIS and TCT have a relationship with the following tests for mobility: the gait subscale of Tinneti Performance Oriented Mobility Assessment, Functional Ambulation Category, 10-meter Walk Test in stroke survivors [11]. And the PASS-TC is correlated with Barthel Index and balance subscale of Fugl-Meyer Assessment [12].

As the results of these previous studies, sitting balance, trunk control and mobility are correlated with each other [13-17]. However, if SBS is a tool for evaluating the balance of patients who have remarkably limited gait or have difficulty maintaining the standing balance, it is necessary to verify whether it has discrimination as a sitting balance evaluation tool that can predict whether a person can actually walk.
Therefore, the aim of this study is to confirm the sitting balance and trunk control measured using the evaluation tools, such as SBS, TIS, TCT and PASS-TC, and mobility have meaningful relationship of sub-acute stroke survivors. In addition, this study is to examine whether the sitting balance and trunk control can predict mobility.**”**

3) Please state your research hypothesis. If you want to reveal scale properties in an exploratory manner, please insist on doing so. I don’t understand why mobility is suddenly mentioned at the end of Introduction.

Answer) Thank you for your kind comments. The Introduction section was revised why sitting balance and trunk control are important in stroke rehabilitation. Also, sitting balance and trunk control have to be restored to increase the mobility of stroke survivors, and the variances are important to inform patients and their family of possible levels of mobility recovery. All of changed contents are highlighted in red.

#2. Again, you will also need to answer the following question.

1) “Most importantly, I don’t see the clinical significance of predicting current TUG in a patient with measurable TUG. What is important is not predicting TUG, but that the mobility to be assessed by TUG can be explained a lot by sitting balance?”

Previous studies have proposed the importance of trunk/sitting balance performance to predict future TUG (not the current TUG).

Answer) Thank you for your kind comments. We totally agree with your opinion. When comparing the siting balance and trunk mobility based on the current mobility level in Table 1 of this study, it was confirmed that there was a significant difference. In addition, since TUG was used as a variable representing the mobility level of stroke patients in previous studies, mobility was evaluated through TUG assessment in this study. The Introduction section has been revised for a reasonable explanation of this point. All of changed contents are highlighted in red.

#3. Related to the above, the overall structure of the introduction section could be further improved. The introduction flow sounds like it emphasizes the advantage of SBS, but is doesn’t seem to be connected to the hypothesis or purpose. Isn’t the flow that SBS is supposed to reflect TUG more than TCT, TIS, or PASS-TC?

Answer) Thank you for your kind comments. This study attempted to find the most appropriate evaluation tool to predict mobility by using various methods to assess the siting balance and trunk control. In the rehabilitation process of a stroke patient, predicting the level of mobility that the patient can recover is important for the patient's motivation to participate in rehabilitation and the mental health of the patient's family. According to the results of this study, it was confirmed that the predictability of TUG (mobility) through SBS was the highest compared to TCT, TIS or PASS-TC. We added this point in Introduction section, all of changed contents are highlighted in red.

If you want to emphasize SBS, the significance of the description of BBS and FRT before it appears to be unimportant. Is SBS an alternative to BBS and FRT? It might be more appropriate to emphasize mobility first instead.

For example.

1. General remarks: stroke and their balance and gait ability

2. Mobility: the significance and necessity of predicting mobility

3. Current status of indices proposed for this purpose

4. Validity of the SBS

5. Hypothesis and purpose

Answer) Thank you for your kind comments. First of all, we rewritten the Introduction section again as you suggested. The Introduction section was corrected as the sequence of the subtitle.

In particular, “2. Mobility“ is important because they determine the significance of this study.

Again, is there a need to bother predicting the current TUG?

If you change the introduction, please reorganize the discussion with the corresponding text.

Answer) Thank you for your kind comments. It is believed that there are many ways to measure the mobility of stroke patients. In this study, however, we assessed the mobility through the TUG because it shows significant validity and reliability in the rehabilitation process of stroke patients. Also, the TUG is a measurement tool that can be used without much difficulty in clinical practice.

**Minor comments**

#4. Title

It may need to be modified to meet the purpose of the text.

Consider whether it is appropriate to use the term "predictive" for current mobility.

For example, it might be as simple as "the relationship between trunk/sitting balance performance and mobility".

Answer) Thank you for your kind comments. We corrected the Title of this study as you suggested. All of changed contents are highlighted in red.

**“The relationship between sitting balance, trunk control and mobility with predictive for current mobility level in survivors of sub-acute stroke”**

#5. Abstract

1) Your purpose is the same as the title. Isn't the main message to identify what can predict mobility level?　What is the significance of looking at the relationship between clinical indicators?

Answer) Thank you for your kind comments. As per your comment, we have revised the description in abstract section. All of changed contents are highlighted in red.

**“ABSTRACT**

**Purpose:** To investigate the relationship between sitting balance, trunk control, and mobility, as well as whether the sitting balance and trunk control can predict mobility level in sub-acute stroke survivors.

**Design:** A observational and cross-sectional study.

**Methods:** Fifty-five hemiplegic stroke survivors were participated in this study. The Timed Up and Go Test (TUG) was used to estimate mobility, and the Sitting Balance Scale (SBS) was used to examining sitting balance. The Trunk Impairment Scale (TIS), Trunk Control Test (TCT), and Postural Assessment Scale for Stroke-trunk control (PASS-TC) were used for examining the trunk control.

**Findings:** The TUG is significantly correlated with SBS (r = -0.78), TIS (r = -0.76), TCT (r = -0.65), and PASS-TC (r = -0.67). In addition, the receiver operation characteristic (ROC) curve showed as cut-off value of SBS as >28.5, TIS > 16.5, TCT >82, and PASS-TC >10.5. The area under the ROC curve in each of the four tests is moderately accurate for predicting the mobility of sub-acute stroke survivors (.84 ~ .90) (0.7 < AUC ≤ 9 (moderate informative)).

**Conclusion:** The SBS showed the highest correlation for mobility using TUG in the hemiplegic stroke survivors. Also, SBS was revealed as the most dominant examination tool predicting the mobility by TUG, it can be explained the sitting postural balance is the variable predicting the mobility in survivors of sub-acute stroke.**”**

2) Is there a specific reason to focus on the sub-acute phase? If not, this information should be in Methods.

Answer) Thank you for your question. Generally, most stroke patients in acute stage may be still medically unstable and have not started rehabilitation in earnest. And, in chronic stroke patients, recovery was almost advanced, and the recovery rate was slow or nearly reached a plateau. Thus, this study investigated for sub-acute stroke patients. Subsequently we tried to verify the relationship between subject's sitting balance and trunk control and mobility, this study was conducted on sub-acute stroke patients. All of changed contents are highlighted in red.

3) Methods:

The sample size of 65 participants in methods is misleading, and the actual number of participants (55) needs to be indicated. There is no information at all to process the predictions. It is just a list of scales, but it is not a research method.

Answer) Thank you for your kind comments. Sixty-five sub-acute stroke survivors were participated and 10 subjects dropped out, thus the results of 55 stroke survivors were analyzed. In Participants section of Method, the changed contents are highlighted in red.

Findings:

Isn't ROC to “predict the TUG”? Results about predictions are not describe in the methods.

Please standardize the notation of 0 ( .84 or -0.78).

Answer) Thank you for your kind comments. As per your comment, we have added the description in methods section of abstract. All of changed contents are highlighted in red.

Conclusion:

“Sub-acute stroke survivors” is repeated in the Conclusion. Other redundant expressions are also noticeable

Answer) Thank you for your kind comments. As per your comment, we have revised the expression. All of changed contents are highlighted in red.

#6. Introduction

You describe the relationship between each indicator, but I don't understand what you are trying to explain. Is the purpose of your research to verify the validity of the scale? It is not appropriate to say that you examined the relationship because it is unknown. Please discuss what you are trying to say by examining the relationship.

Answer) Thank you for your kind comments. As per your comment, we have rewritten the Introduction section to make it clearer that the order and content of manuscripts have been changed to further emphasize the meaning of SBS. All of changed contents are highlighted in red.

#7. Introduction

What does the percentages in the relationship sentences in TIS and TCT stand for?

Answer) Thank you for your kind comments. The percentage is the relationship with mobility assessment tool, such as gait subscale of Tinneti Performance Oriented Mobility Assessment, Functional Ambulation Category, 10-meter Walk Test. We deleted the percentage of each tools to clarify the meaning of the sentences in Introduction.

#8. Introduction

Please do not abbreviate the BI of the first appearance.

Answer) Thank you for your kind comments. Activities of daily living were deleted due to lack of meaning for the purpose and results of this study. In addition, the order and content of the paragraphs have been revised to clarify the necessity of this study.

#9. Discussion

The discussion is redundant (e.g., repetition of the same terms, repetition of the same kind of arguments). Please organize your arguments.

Answer) Thank you for your kind comments. As per your comments, we have rewritten the discussion section as follows. All of changed contents are highlighted in red.

**“Discussion**

The sitting balance and trunk control can be impaired after stroke, it is important to improve it for significant rehabilitation of stroke survivors [11, 17]. In particular, hemiplegic side of trunk can make the impairment of sitting balance and trunk control [26], these factors can limit the mobility of stroke survivors. The results of the present study demonstrated that mobility impaired group had significantly decreased mobility (TUG), sitting balance (SBS), and trunk control (TIS, TCT, PASS-TC) than mobility available group. Trunk stability exercise improve trunk control, dynamic sitting balance, standing balance gait and activities of daily living in subacute stroke survivors [18]. In the results of this study, it was found that the sitting balance and trunk control level were low in patients with subacute stroke with low mobility level by using TUG. If the sitting balance and trunk control level decrease, it is difficult to achieve normal gait because the center of gravity cannot be maintained normally. Thus, the decreased trunk function makes it difficult to perform normal gait that leads the lowered mobility level.

Through the present study, the SBS demonstrated the highest correlation for mobility using TUG. It is thought the SBS contains the postural balance compared to the other tests, and have items center on the anticipatory demands of upper or lower extremity movement.
The present study, cut-off values of SBS, TCT, PASS-TC and TIS were used as a reference point for estimating the mobility level. In these examinations, it was confirmed that the AUC was moderately accurate (curve area = 0.84 ~ 0.90) [25]. In addition, for the first time, we presented a selection criterion for predicting mobility level. Specificity was acceptable in all four examinations (78% to 87%), specificity (71% to 85%) and positive predictive value (90% to 94%), negative predictive value (57% ~ 68%) was somewhat low.
As a result of this study, prediction of mobility level for stroke survivors who have 4 months of disease duration and minimal to moderate paralysis was found that SBS, TIS, TCT, and PASS-TC are excellent screening methods and show positive predictive value because of sensitivity, specificity and screening test. The negative predictive value of these tests was found to be somewhat low. As in previous studies [10, 23], the study classified that if the time to perform a TUG test is greater than 20 seconds, It’s a mobility available group that can walk and if the time to perform a TUG test is equal or less than 20 seconds, it's a mobility impaired group that can’t walk. Specifically, the stroke survivors who needed considerably short time for examining TUG, have inappropriate compensatory strategy and use assistive device but independent mobility is possible may not be included. Therefore, more survivors would be required and a more quantitative approach is needed for the classification criteria of mobility level. However, in this study, the selection criteria for discriminating the mobility level in the sitting balance (SBS) and trunk control (TIS, TCT, and PASS-TC) is that sitting balance and trunk control should be maintained before functional movement and restoration of the preceding postural control and mobility [27].
The relationship between SBS and TIS for the elderly was also verified in various conditions (acute phase patient nursing r = 0.92, rehabilitation nursing r = 0.89, intensive nursing r = 0.88, home nursing r = 0.60); both variables are very closely related [10]. In contrast, TCT and PASS-TC consist of only four to five items that examine mobility in bed. Since there is no item that can affect the TUG, it can be seen as a low examination of discrimination tool. In particular, TCT is not capable of qualitative examination of trunk movement [28], and there was a moderate correlation with the trunk muscle strength using the dynamometer [29].

In the present study, it was confirmed that SBS has the highest predictive validity in discriminating mobility level. Unlike TIS, TCT, and PASS-TC, the SBS is consisted of controlling the upper and lower trunk and coordination, as well as a specific task item for examining the comprehensive dynamic balance capability, required for sit to stand task and mobility. This was also confirmed in the factor analysis (except for TCT and PASS-TC) affecting TUG. In the Rasch analysis, static section among TIS items was not suitable because of the ceiling effect in subacute and chronic stroke survivors, this is also seemed to have not affected the power of discrimination of static section [30, 31].
This study have several limitations. First, the sample size in the mobility impaired group was relatively small compared to number of mobility available group. In addition, the age group of the participants was relatively low. And the participants in TUG test were performed based on a specific point in time, thus, it may be limited in its adaptation to all stroke survivors. The second, Medley et al and Thompson reported that SBS is the most objective clinical examination method because it focuses on comprehensive balance examination rather than trunk control examination of TIS [24, 28]. Therefore, in SBS, which is traditionally used, it may be an appropriate examination tool to determine the discrimination of sitting balance and mobility in stroke patients with significantly impaired balance and gait performance. Future studies will need standardization to determine the superiority of sitting balance using convenience sampling of normal individuals and stroke patients. Lastly, the use of new examination tools will require research that reflects the psychological characteristics (sensitivity, specificity, response rate) of patients with various neurological disorders.”

#10. Discussion

Regarding the sentence "For the first time, we presented a selection criterion for predicting mobility level", as pointed out in the intro, I did not understand the need to show the predicted TUG for those who are capable of TUG using other indicators. Please include this reason and emphasize it again in your discussion. Otherwise, the significance of the study shown “for the first time” will not be conveyed.

Answer) Answer) Thank you for your kind comments. As per your comment, we have revised the discussion section as above.

#11. Discussion

“It's a mobility available group that can walk and if the time to perform a TUG test is equal or less than 20 seconds, it's a mobility impaired group that can't walk”.

What is the point you are trying to make with these related sentences? Is it a repetition of the methodology?

Answer) Thank you for your kind comments. It was to find out whether sitting balance and trunk control are correlated with mobility (TUG) as the result of several previous studies. So, the result whether there are differences between sitting balance and trunk control according to mobility level is written in Table 1, and this is described in the Discussion section.

#12. Conclusion

The conclusion does not receive your purpose. The objective to lead this conclusion is “does the sitting balance correlate with current mobility?” I'm not sure if “prediction” is the right term, but it is common for linear prediction to hold if there is a correlation.

Isn't the basic argument and conclusion not the validity of the predictions, but how the sitting balance relates to mobility?

If you want to argue for predictive validity, I think the title and purpose should reflect “the validity of the sitting balance that predicts the current TUG”.

If the latter, sitting balance is highly correlated with mobility, so it is important to focus on sitting balance in sub-acute stroke.

Answer) Thank you for your kind comments. As per your comment, we have revised the conclusion section as follows.

**“Conclusion**

On the present study, the SBS showed the highest correlation for mobility using TUG in sub-acute stroke survivors. In addition, it was found that the SBS is the most appropriate examination tool for predicting the mobility by the TUG in sub-acute stroke survivors. Thus, it may be suggested the sitting balance and trunk control can be important for future mobility. In early stage of rehabilitation of sub-acute stroke patients, it may be focused on improving the sitting balance and trunk control.**”**

Additional comment

There are sentences that need to be consistent with the time period as a whole. In particular, the report of previous studies should be shown in the past tense.

Answer) Thank you for your kind comments. As per your comment, we have checked and revised the whole manuscript.
