# Supplementary material for: The relationship between sitting balance, trunk control and mobility with predictive for current mobility level in survivors of sub-acute stroke
Source: PLoS One. 2021 Aug 5;16(8):e0251977. doi: 10.1371/journal.pone.0251977 (PMC8341576; doi:10.1371/journal.pone.0251977)
Supplement: Attachment — Submitted filename: Response to reviewers comments.docx [file pone.0251977.s005.docx]

**Response to comments**

4. Have the authors made all data underlying the findings in their manuscript fully available?

The [PLOS Data policy](http://www.plosone.org/static/policies.action#sharing) requires authors to make all data underlying the findings described in their manuscript fully available without restriction, with rare exception (please refer to the Data Availability Statement in the manuscript PDF file). The data should be provided as part of the manuscript or its supporting information, or deposited to a public repository. For example, in addition to summary statistics, the data points behind means, medians and variance measures should be available. If there are restrictions on publicly sharing data—e.g. participant privacy or use of data from a third party—those must be specified.

Reviewer #1: Yes

Reviewer #2: No

6. Review Comments to the Author

Please use the space provided to explain your answers to the questions above. You may also include additional comments for the author, including concerns about dual publication, research ethics, or publication ethics. (Please upload your review as an attachment if it exceeds 20,000 characters)

Reviewer #1: (No Response)

Reviewer #2: Abstract
Is this sufficient to describe the Methods? It doesn't seem to correspond to Finding section.
The following is my previous comment. I will present this again.

Findings:
Isn't ROC to “predict the TUG”? Results about predictions are not describe in the methods.
Please standardize the notation of 0 ( .84 or -0.78).

Answer) Thank you for your kind comments. A sentence corresponding to the result of this study was added in Methods of Abstracts. In addition, we corrected the notations for consistency. We apologize for repeating the same mistake.
